# Abstract of Meteorological Observations for February, 1856, and for the Winter Quarter Ending February 29th, 1856, Made at Philadelphia, Pa.

**Published:** 1856-04

**Authors:** James A. Kirkpatrick


					﻿Barometer. Thermom. '	“	-----------------------------------------_________________
^.^2 <■ -----------------------------------------
rS O	1856	„ ., M.ean Mean Re!. Forceof Dew ’Rain &	Remarks	§
K	Februarv 2raily L "y E?,ly Paily Huraid- .Vnpor Point ’Melted Prevailing	Remarks.
e	reoruary. Mean Range. Mean Range 2 p.M. 2 P.M. 2P.M. Snow. Winds.5	“
O' 2	s	Inches.	Inches-	De?'	De8-	Hunds.	Inches.	Deg.	Inches.	Prints’	------------------------
iQ	1	29.598	.280 30.5 9.5	75	.163 29.5	SW	vr„ •	„ .	S
oo	~ a	2	29.601	.072 22.0 12.5	63	. 089 15 8	WNW	Morning	and afternoon	cloudy; evening clear.
<-i o	isj	3	29.734	.133	8.5	13.5	60	.043	-0 1	w ’	ni' Ddawar& Riwr cto«ed with ice,	as far	as Chester.
o’S I"	2	4	29.790	.056	9.0	2.8	64	.054	4.0	w'
&	30.063 .273 12.5 3.5	59	.055	5.2	W’ 012?
e	H	6	30.319 .384 17.8 5.3	73	.090 16.1	WSW Kn. a «	5
~	~ S <	7	29.791 .525 31.2 13.3	90	.183 32.3	0.738 rVar 1	• S d aftern0011 olear > evening cloudy. Barometer highest 30.382. S
M	8	29-715 -086 33.5 5.7	80	.170 30.5	0.052 NE’J cinmlv? n°W unt11 a’ A-M'! then rain till 3, P.M.; evening cloudy. £
r_? 00 S	9	29.784 .091 26.3 7.2	55	.089 15.8	eVar’l vf1°,^;y’aft®rn°on and ®vening snoto; stopped during night.	<x»
r—l S ~	10	29.987 .204 26.8 6.8	62	.127 23.7	(Vari	and afteJnooncloudy! 8 j, A. M., a slight sprinkling of snow; eve- ®
-uS	11	29.687 .300 37.3 10.3	60	.172 30.8	Sw'	hazy; aftern00n cl°udy; evening clear.	[ning clear.] 3
.	12	29-418 -451 27.7 17.0	40	.071 10.9	SW ’ Mnrnhr’a nnH rt	,	2
^31.	13	29.943 -525 8.2 19.5	59	.043 -0.1	NW	aad afternoon cloudy; evening clear. Barometer lowest 29.139. <S
<71 J> ~	14	30.022 .109 12.0 4.2	59	.055	4.4	W ' r>“X- mb	.	,	W
§	=°	15	29.618 .401 27.7 15.7	52	.102 18.8	0-011 SW. Cloudv'- l^p’T^R^p^2^'	2
•2	§	16	29.227	.391	35.2	7.5	70	.183	32.3	(Var )	Cloudv’• mSrni^’f 8*’ P‘ M-’ Sn°W-	~
S3	§	71	5	17	29.420	.236	18.8	16.7	35	.033	-5.9	W.	Moroni and afte^T 1 a	d
s>	£	_	18	29.616	.196	17.8	6.3	61	.078	12.9	NW.	c °™lng and afternoon cloudy; evening clear.	.S
20	29718	231	318	12 0	46	’117	218	w^w	^orning	clear5 afternoon and evening cloudy.	£
O	21	29:629 :089 36.7 4 8 tt ^55 28 3	Tw “or“nS and ^noon cloudy; evening clear.	g
22	29.683	.06-2	39.8	3.5	40	.136	25.2	WSw	Morning	hazv^ft"0011 cloudJ; evening clear.	£
26	29.781	.193	34.3	2.5	63	.144	26.6	Nw	Cloudv	a 68
______29	42	.100 18^3	'w0	&X
S Means ) 1856 29.731 .228 26.7 8.1	60	.112 18.4 1.128 N. 90° W 44-100	— ———————————	£
J, » X for > ____________________________________________________________________________ eH □
Feb., >5 yrs, 29,867 .194 31.2	6.9	70	.151	26.3	2-992 N. 68° 57'W.33-100.	~ ®
forthe^ —29-84° ~232 29'3	7’°	74	134	22-4	9'5°2 N. 73° 40'”w 37-100.	g ®
S winter S 5 yrs. 29914	—-g-—
-O -	S
-____________________________________________ *•
‘	-----------------—-------------------------------------------------------------.g
				

